# Germanium/perovskite heterostructure for high-performance and broadband photodetector from visible to infrared telecommunication band

**DOI:** 10.1038/s41377-019-0218-y

**Published:** 2019-11-21

**Authors:** Wei Hu, Hui Cong, Wei Huang, Yu Huang, Lijuan Chen, Anlian Pan, Chunlai Xue

**Affiliations:** 1 Key Laboratory for Micro-Nano Physics and Technology of Hunan Province, School of Physics and Electronics, Hunan University, Changsha, Hunan 410082 China; 2 State Key Laboratory on Integrated Optoelectronics, Institute of Semiconductors, Chinese Academy of Sciences, Beijing, 100083 China; 3 Center of Materials Science and Optoelectronics Engineering, University of Chinese Academy of Sciences, Beijing, 100049 China; 4 Key Laboratory for Micro-Nano Physics and Technology of Hunan Province, College of Materials Science and Engineering, Hunan University, Changsha, Hunan 410082 China

**Keywords:** Silicon photonics, Green photonics

## Abstract

A high-performance and broadband heterojunction photodetector has been successfully fabricated. The heterostructure device is based on a uniform and pinhole-free perovskite film constructed on top of a single-crystal germanium layer. The perovskite/germanium photodetector shows enhanced performance and a broad spectrum compared with the single-material-based device. The photon response properties are characterized in detail from the visible to near-infrared spectrum. At an optical fibre communication wavelength of 1550 nm, the heterojunction device exhibits the highest responsivity of 1.4 A/W. The performance is promoted because of an antireflection perovskite coating, the thickness of which is optimized to 150 nm at the telecommunication band. At a visible light wavelength of 680 nm, the device shows outstanding responsivity and detectivity of 228 A/W and 1.6 × 10^10^ Jones, respectively. These excellent properties arise from the photoconductive gain boost in the heterostructure device. The presented heterojunction photodetector provides a competitive approach for wide-spectrum photodetection from visible to optical communication areas. Based on the distinguished capacity of light detection and harvesting from the visible to near-infrared spectrum, the designed germanium/perovskite heterostructure configuration is believed to provide new building blocks for novel optoelectronic devices.

## Perovskite-germanium layers for broadband light detection

A device made from germanium and perovskite layers can detect light in the visible and near-infrared ranges, showing potential for use in a wide range of applications, including in optical communications and next-generation optoelectronics. This heterojunction photodetector fabricated by Chunlai Xue of the Chinese Academy of Sciences and colleagues overcomes problems in single-material photodetectors, which are unable to detect a broad range of light. Recent research into various combinations of semiconducting materials for heterojunction photodetectors has led to devices with poor sensitivity to light or that require a high working voltage. Adding a layer of methylammonium lead triiodide perovskite to a layer of germanium resulted in a highly sensitive photodetector at the optical fibre communication wavelength of 1550 nm (near-infrared range) and the visible light wavelength of 680 nm.

## Introduction

A photodetector (PD) is an optoelectronic device widely used to convert light signals into electronic outputs. The photon response spectrum of a PD is critical for its detection application. This property is generally determined by the specific bandgap of a semiconducting active layer applied in a device^1–3^. A broadband photodetector, which can detect from visible (Vis) to infrared (IR) light, is particularly important in the commercial applications of imaging sensors, optical communication, environmental monitoring, and civil engineering^4–6^. However, a single semiconductor is hardly able to achieve a broader response spectrum as an active layer in a photodetector. For example, the inorganic semiconductor germanium (Ge) has been applied to construct a key component of photodetection in optical interconnection and optoelectronic integrated circuits (OEICs). It has unique optoelectronic properties at the IR telecommunication band and great process compatibility with complementary metal-oxide-semiconductor (CMOS) techniques^7,8^. Unfortunately, the results by far indicate that germanium has extremely poor response performance among the Vis light spectrum. The reasons for the shortcoming mainly lie in the short Vis light penetration length and low photogenerated carrier collection efficiency in germanium film (called the "dead region effect"). These limitations hinder it in the development of Vis-light communication, not to mention broadband absorption applications^9–12^.

To overcome the challenges mentioned above, much efforts have been made to construct heterojunction devices in recent decades. Many different kinds of materials, including layer materials such as graphene^13^ and MoS_2_^14^, inorganic and organic semiconductors such as PbS^15^, ZnO^16^, PDPP3T^17^ and Si^18^, have been explored together to capture more incident photons. For example, the graphene/Bi_2_Te_3_ heterostructure photodetector shows a broadband response from 532 to 1550 nm^13^. However, it has low responsivity over the response spectrum, especially in the NIR spectrum (0.22 A/W at 1550 nm) due to weak optical absorption of the layered graphene. The perovskite/MoS_2_-based photodetector can detect visible light only due to the bandgap limitation of the two layers, and shows a peak responsivity of 68 A/W at a wavelength of 514 nm^14^. Moreover, a high temperature over hundreds of degrees is typically used for the deposition of ZnO, TiO_2_ and Si film, which would cause critical damages to the underlying layers or substrates. Based on the research works conducted so far^13–23^, the limited response spectrum, low performance, high working voltage and incompatibility of the procedure at low temperatures have become major concerns for the heterojunction photodetector construction. These disadvantages also strongly hamper their widespread commercialization applications.

Recently, a series of solution-processed organic-inorganic hybrid perovskite has attracted extensive attention in the research area of optoelectronic devices. Methylammonium lead triiodide (CH_3_NH_3_PbI_3_) is the most representative one among these materials. The CH_3_NH_3_PbI_3_ perovskite thin film can be easily synthesized, which has excellent advantages such as a direct bandgap^24^, long charge carrier diffusion length^25^, low recombination rate and high absorption coefficient in the Vis light range^26^. Its application in the areas of optical amplification^27^, nonlinear optical areas^28^, and light-emitting diodes^29^ has been studied. In the last two decades, the CH_3_NH_3_PbI_3_ thin film has been mostly explored as light harvester of a solar cell^30,31^, which has an energy-conversion efficiency of over 25%^32^. The perovskite thin film has also been explored as an active layer in photodetectors with vertical (photovoltaic type) and lateral (metal-semiconductor-metal type) device architectures^33–43^. The devices exhibited good photo response properties under Vis light illumination. However, they cannot absorb the photons in the IR spectrum. The reported photodetectors show a cut-off wavelength of 780 nm due to the bandgap limitation of the perovskite absorber^37–40^. This means that the CH_3_NH_3_PbI_3_ perovskite features strong absorption in the Vis light spectrum and high transparency in the near-IR spectrum. Therefore, the perovskite material is an appropriate candidate, being constructed with germanium, which is a heterostructure photodetector aiming to match the Vis-to-IR broad photo response requirement.

## Results

**Fig. 1:**
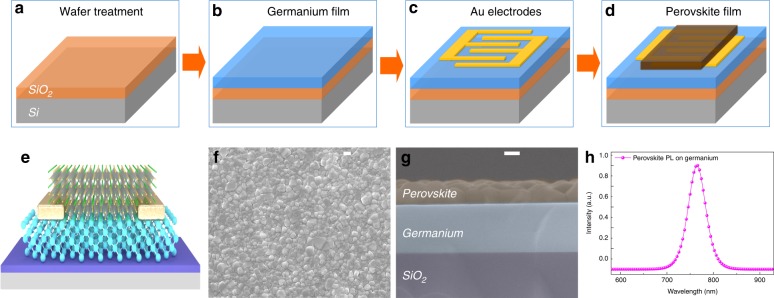
Schematic illustration of the germanium/perovskite heterojunction device fabrication process. **a** A cleaned SiO_2_/Si substrate. **b** A germanium layer growth by MBE. **c** Au electrode deposition on the substrate. **d** Perovskite layer construction by the vapor-solution method. **e** Three-dimensional diagram of the heterojunction photodetector. **f** Top-view and **g** cross-sectional SEM image of the achieved heterostructure device (scale bar = 200 nm). **h** Steady-state photoluminescence spectrum of the constructed perovskite film.

A perovskite/germanium heterojunction photodetector with excellent photo-response properties has been successfully fabricated. As shown in Fig. 1, the fabrication process of a germanium/perovskite heterojunction photodetector is schematically exhibited. First, a germanium layer with a thickness of 300 nm is grown by a solid-source molecular beam epitaxy (MBE) technique. The germanium on insulator (GOI) samples are fabricated by a wafer bonding process^44,45^. Second, interdigital gold electrodes are formed by using a thermal vapor deposition method. The channel length and width are defined by a shadow mask. Third, a PbI_2_ layer is deposited on top of the sample, the thickness of which is monitored precisely by a quartz crystal oscillation. Then, a drop of CH_3_NH_3_I solution (in isopropanol) is spin-coated onto the as-constructed PbI_2_ film. Finally, the heterojunction photodetector is constructed via an annealing process performed for 30 min at 100 °C. More details can be found in the experiment section and our previous works^43^.

Three-dimensional diagram of a perovskite/germanium heterojunction photodetector is shown in Fig. 1e. A top-view scanning electron microscopy (SEM) image of the perovskite layer is illustrated in Fig. 1f. A cross-sectional SEM image of the device is presented in Fig. 1g. These images present a compact and pinhole-free perovskite film fully covering on the germanium layer. Figure 1h shows the steady-state photoluminescence (PL) spectrum of the constructed perovskite layer. The peak wavelength is located at 765 nm, which is consistent with previous results^27–31^. For comparison, devices based on pristine perovskite and germanium active layers have been fabricated. The optical and electronic properties of these obtained photodetectors were further performed under the same conditions as those of the heterojunction devices. All these measurements were characterized in air without being encapsulated at room temperature.

In a germanium-based IR photodetector, a considerable portion of incident IR light is reflected from the germanium surface. The germanium active layer has a relatively high refractive index (*n*_*G* = _4.0) and a low absorption coefficient^44,45^. The light off the device suppresses photocurrent formation, which is undesirable in photon-absorption-based devices. Use of an antireflection (AR) coating is an efficient approach to reducing light reflection and maximize transmission into the active layer^46–48^. As we know, the CH_3_NH_3_PbI_3_ perovskite layer is transparent at the telecommunication band due to its bandgap limitation of 1.5 eV. Meanwhile, it has a relatively lower refractive index (*n*_*p*_) of 2.3 compared with that of germanium^49,50^. These advantages suggest that the perovskite material is an appropriate AR coating for germanium-based high-performance photodetectors. To take advantage of both materials to overcome the challenges discussed above, we introduce a germanium/perovskite heterojunction based on the following considerations. First, the perovskite and germanium films are used as absorbers for the effective absorption of Vis and IR light, respectively. Second, the top perovskite layer is applied as an AR coating with regard to the IR spectrum due to its lower refractive index and wider bandgap than those of germanium. At a wavelength of 1550 nm, the thickness of the perovskite AR coating is optimized to achieve the lowest reflection in the photodetector. Third, type-I energy band alignment forms in the germanium/perovskite heterojunction. It can be noted that there is a large band offset between the conduction and valence bands of the two semiconducting materials. This offset easily produces a charge carrier transport from the perovskite into the germanium. The ultra-high carrier mobility in germanium results in a photoconductive gain boost in the heterojunction photodetector. Above all, the germanium/perovskite hetero-structure configuration not only benefits the broadband photodetector but also provides a possible method of developing novel optoelectronic applications. The mechanism of the germanium/perovskite heterojunction photodetector is described in detail below.

**Fig. 2:**
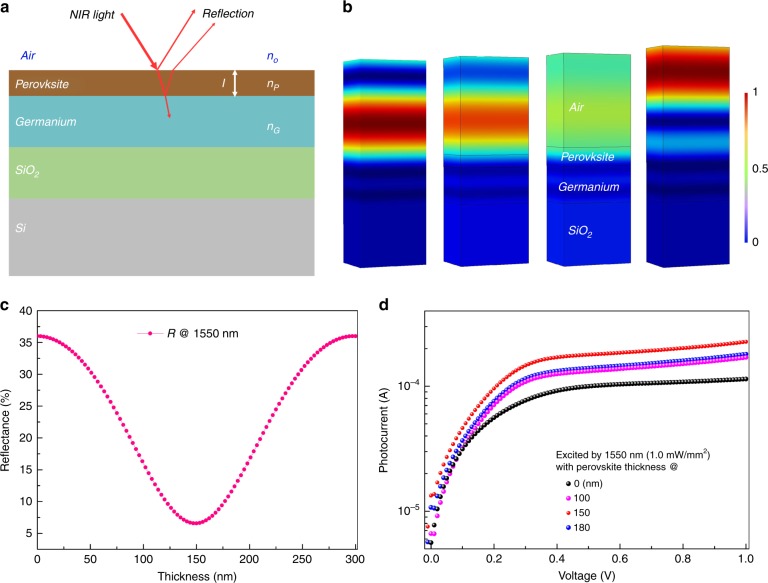
Antireflection coating perovskite film optimization on germanium at a telecommunication wavelength of 1550 nm. **a** Reflection model of incident IR light from both interfaces of the device. **b** Simulation of the optical field distribution for the device with 0, 100, 150, and 300 nm perovskite AR-coated layer. **c** The variation in reflectance with the perovskite thickness at a wavelength of 1550 nm. **d** *I*_ph_*-V* curve comparison for the obtained photodetectors for different perovskite AR coating thicknesses.

As shown in Fig. 2a, the incident IR light is partly reflected off the device because of the refractive difference between the semiconductor layer and air. Based on the fundamental principles of optics^51^, the reflectivity portion is determined by the perovskite AR coating thickness (*l*) and the incident wavelength (λ). To clarify the function of the perovskite AR coating, a calculation is first carried out to evaluate the relationship between the reflectivity and perovskite film thickness. As shown in Fig. 2c, the basic reflectance off surface of the pristine germanium layer is ~36% at a telecommunication wavelength of 1550 nm. The value becomes significantly lower when a perovskite layer is coated onto the germanium as the AR coating. When the perovskite film thickens to approximately 150 nm, the lowest reflectance of 7% is obtained. Then, the value rises slightly and reaches the highest value (35%) as the AR film thickness exceeds 300 nm. The images of the optical field distribution in the bilayer device are also simulated and presented. The full 3D electrodynamics finite element method (FEM) simulations are performed using the COMSOL Multiphysics software. Figure 2b and Fig. S1 show the simulation results for the photodetector with different AR coating thicknesses (0, 100, 120, 150, 180, and 300 nm) at a wavelength of 1550 nm. It can be seen that a 150 nm AR coating exhibits the lowest IR photon loss. The theoretical results predict that the lowest reflectance can be achieved with an optimized perovskite AR thickness about 150 nm. Meanwhile, the enhanced transmit effect in the IR spectrum is observed, as shown in Fig. S2. This effect indicates that a perovskite AR coating with an optimized thickness at a wavelength of 1550 nm can effectively reduce the photon loss in the range of IR spectrum. Based on the analysis above, a batch of heterojunction photodetectors with different of perovskite AR coating thicknesses are constructed and characterized. The perovskite thickness can be precisely controlled in the first step during the PbI_2_ layer fabrication process. The cross-sectional SEM figures of the devices are shown in Fig. 1g and Fig. S3 for different perovskite thicknesses. The photocurrents (*I*_ph_), defined as the current difference between illumination and darkness, are presented in Fig. 2d under an incident wavelength of 1550 nm. Compared with the pristine germanium device, *I*_ph_ increases obviously in the perovskite AR-coated device. The highest *I*_*ph*_ value is obtained in a heterojunction device when the perovskite AR coating thickness is approximately 150 nm. The experimental results also match the theoretical predication discussed above. Figure S4 presents the *I*_ph_*-V* comparison for the pristine germanium and AR coating optimized device. The higher *I*_ph_ of the perovskite/germanium heterojunction device is obvious.

**Fig. 3:**
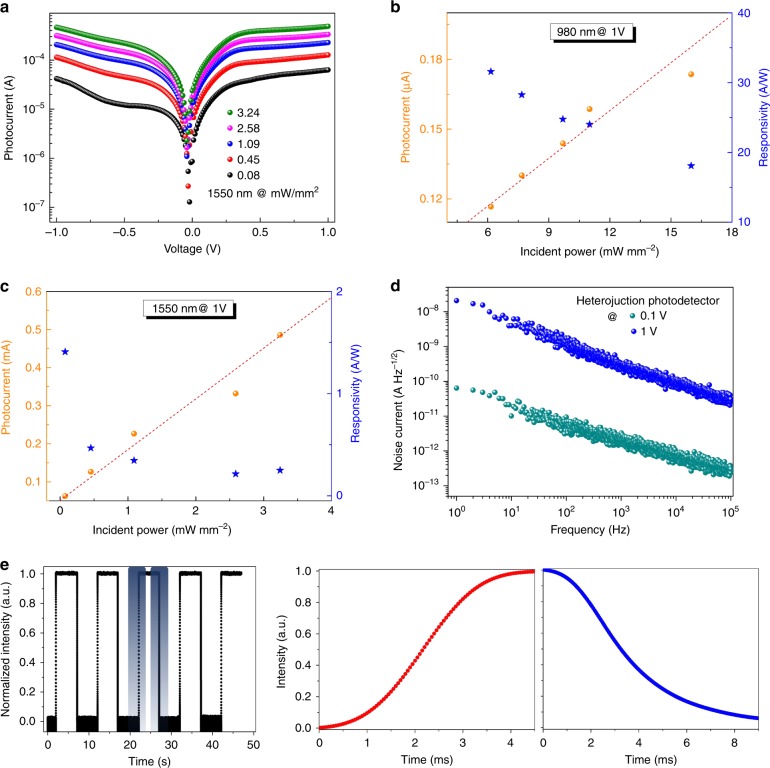
The performance of the optimized heterojunction photodetector in the infrared spectrum. **a** Device *I*-*V* curves and **b** *I*_*ph*_ and *R* values under an illumination wavelength of 1550 nm. **c** Noise currents of the heterojunction device, varying with the frequency (0.1 and 1 V). **d** *I*_ph_ and *R* of the device under an incident wavelength of 980 nm. **e** Device response speed under an incident wavelength of 1550 nm. The zoomed-in views of the rise and decay versus time correspond to start times of 22.110 and 27.029 s, respectively.

Meanwhile, the photodetector performance is characterized at an optical communication wavelength of 1550 nm. Figure 3a shows the illumination-power-dependent *I*_ph_*-V* curves of the device. This figure indicates that the *I*_*ph*_ increase with the applied bias and incident power. Figure 3b (solid circle) exhibits the dependence of *I*_*ph*_ on the illumination power at a bias of 1 V. It shows a good linear relationship as the power density increases from 0.08 to 3.24 mW cm^−2^, which suggests that our heterojunction PD is capable of detecting incident light power over a wide range. The important figure of merits, the photodetector spectral responsivity (*R*) and the detectivity (D^*^) have also been characterized. *R* *=* *I*_ph_*/P*_In_ is defined as the ratio of the device photocurrent to the incident light intensity, in which *P*_*In*_ is the incident optical power. Figure 3b shows the varying trend of *R* (star) with incident power under a bias of 1 V. A maximum value of *R* = 1.4 A W^−1^ at 1550 nm is achieved for the heterojunction photodetector. As far as we know, this is the highest *R* among the reported IR photodetectors to date at a low working voltage of 1 V^7–12^. The responsivity drops significantly as the incident light intensity increases. This result suggests that the photocurrent flowing through the device does not proportionately increase as the incident light intensity increases. The major potential reason is the limited absorption coefficient of the active layer. As the proportion of illumination photons increases, the absorption gradually saturates in the germanium active layer. Then, the photogenerated carrier and photocurrent do not increase accordingly, which results in a decrease in the *R* values. The photodetector performance is presented in Fig. 3d at an incident wavelength of 980 nm. The highest *R* value of 32 A W^−1^ is achieved at a bias of 1 V. Another important parameter, D*, is used to quantitatively evaluate the capability of a detector in weak light detection. It is determined by the responsivity and noise of a photodetector, $D^ \ast = \left( {A\Delta f} \right)^{\frac{1}{2}}R/i_n$, in which *A* is the device effective area and Δ*f* and *i*_*n*_ are the electrical bandwidth and noise current of the device, respectively. Figure 3c shows the noise currents of a heterojunction device at various frequencies. The results for the pristine germanium device are shown in Figure S5. The curves indicate that noise currents decrease as the frequency increases and reach a higher level under a larger voltage due to a higher dark current. At a wavelength of 1550 nm, the D^*^ of a germanium/perovskite photodetector is estimated to be 1 × 10^8^ Jones (cm Hz^1/2^ W^−1^) at 0.1 V, which is better than the reported results for the telecommunication band. The higher values of *R* and D^*^ suggest that our heterojunction photodetector has enhanced performance compared with that of the pristine germanium device. We ascribe the promotion mainly to the introduction of the perovskite AR coating. The perovskite thickness is optimized to reduce the IR photon loss efficiently due to reflection. As more IR photons are transmitted and trapped in the germanium film, more photon-induced charge carriers are generated in the active layer. Consequently, the constructed heterojunction photodetector exhibits higher photocurrent and better performance than those of the pristine germanium detector. Device response speed experiments are also carried out. Figure 3e shows the time-resolved on-off switching behaviors of the photodetector being investigated at an IR wavelength of 1550 nm. The rise and decay time of the heterojunction photodetector are measured to be 2.1 and 5.7 ms at room temperature, respectively, which show higher speeds than those of pristine germanium device^7–10^.

**Fig. 4:**
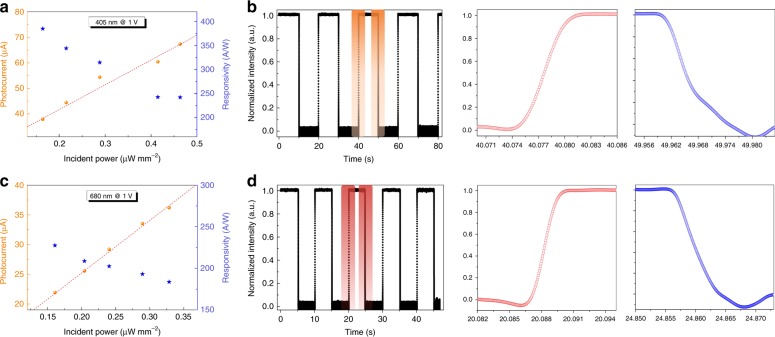
Performance of the heterojunction photodetector in the visible light spectrum. *I*_*ph*_ and *R* of the device under illumination wavelengths of **a** 405 nm and **c** 680 nm. Response speed of the heterojunction photodetector. The rise and decay times of the device under an incident wavelength of **b** 405 and **d** 680 nm.

**Fig. 5:**
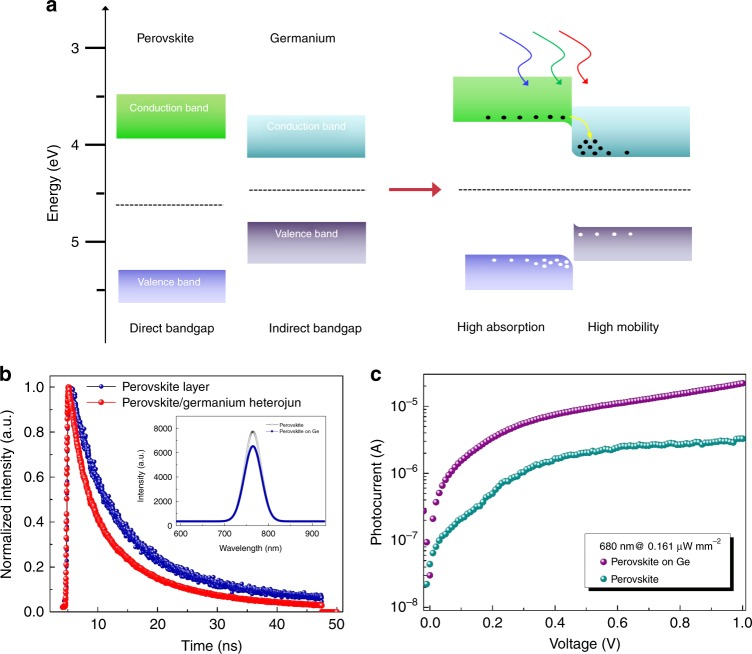
The germanium/perovskite heterojunction photodetector energy band model and optical characterization. **a** Energy-band diagrams for two isolated semiconductors and heterojunction device (type I energy band). **b** Time-resolved PL decay and intensity (inset) comparison for the pristine and hetero-structure perovskite. **c** *I*_ph_*-V* curves for pristine perovskite and heterojunction photodetector.

The heterojunction photodetector performance in the Vis light region has also been characterized. The perovskite layer is chosen to have an optimized thickness of 150 nm, which is approximately two times thinner than the reported devices, including perovskite solar cells and photodetectors^21–26^. Figure 4a, c summarizes the *I*_*ph*_ and *R* values of the device under two typical Vis wavelengths, namely, 405 and 680 nm. Figure S6 shows the *I-V* curve comparison between the pristine germanium and the heterojunction photodetectors. The photocurrents of the heterojunction device are noted to be higher than those of the pristine germanium device. The *I-V* performance of a heterojunction device at different illumination powers is presented in Fig. S7 for varying wavelength in the Vis spectrum. The figures indicate that *I*_*ph*_ increases dramatically with the incident power density. The typical values of *R* and D^*^ under Vis light illumination are obtained for the heterojunction device. For example, a high *R* of 228 A W^−1^ (1 V) at an illumination wavelength of 680 nm is achieved. D^*^ is estimated to be 1.6 × 10^10^ Jones at a 0.1 V bias. These parameters are comparable with the those of the pristine perovskite photodetectors, the active layer thickness of which is approximately two times that of this device^21–29^. Figure 4b, d shows the on-off switching behaviors of the heterojunction device. Under incident light of 680 nm (405 nm), the device rising and decay times are measured to be 1.8 and 5.1 ms (4.1 and 9.2 ms), respectively. The constructed photodetector exhibits higher performance and faster response in the Vis spectrum than those of the pristine device. We ascribe these promotions to bilayer structures based on perovskite and germanium layers in the detector. The device physics based on the energy-band model and optical techniques are studied comprehensively below. Figure 5a shows the energy band of isolated germanium and perovskite materials. The conduction and valence band edges for intrinsic perovskite (germanium) are 3.87 (4.13) eV and 5.45 (4.80) eV^40–44^, respectively. There is a relatively high energy band difference between the two semiconductor materials (0.26 eV for the conduction band and 0.65 eV for the valence band). The Fermi energy difference is 0.18 eV based on the obtained ultraviolet photoelectron spectroscopy (UPS, Fig. S8). At thermal equilibrium, energy band bending occurs at the heterojunction interface due to the requirement of the Fermi level coincident on both sides^52–55^. Then, the conduction band energy difference at the interface is approximately 0.44 eV, arising from band bending, as shown in Fig. 5a. Driven by the large band offset, the photogenerated free electrons in the perovskite layer are transferred easily into the germanium layer. However, the holes in the valence band of the perovskite layer are confined in the valence band due to barrier formation as the band bends downwards. Optical and photoelectronic methods have been carried out to evaluate the effect of interface properties on the device performance. Figure 5b shows the time-resolved PL and decay transient curves for pristine perovskite and on the germanium layer. The experiments are all performed under the same situation. The inset of Fig. 5b shows the PL spectrum with a peak wavelength of 765 nm, which corresponds to the band-edge emission of the CH_3_NH_3_PbI_3_ layer. A slightly lower intensity can be observed for perovskite constructed on germanium. This observation suggests that the density of the photon-generated carrier becomes lower in the perovskite layer constructed on the germanium layer. Based on the curves fitted by the bi-exponential decay function, the carrier lifetime is obtained as shown in Table S1. The fast decay component is associated with trap-assisted recombination, and the slow decay part is ascribed to radiative recombination. In terms of the perovskite on germanium, the obtained decay times are 11.5 and 2.87 ns (fast and slow lifetimes, respectively), which are obviously shorter than the values of 19.0 and 6.1 ns for the pristine perovskite on glass. The PL decay curves suggest that the photogenerated carriers have a marginally faster recombination rate in the perovskite layer of the heterojunction device than that in pristine film. This observation indicates that a number of photogenerated carriers will be delocalized from the perovskite absorption layer. The lower charge carrier lifetime also explains the faster response speed of the heterojunction device. There are two potential reasons for this lower PL intensity and shorter charge carrier lifetime. The first one is that a number of charge carriers are localized by trap states at the interface. The second is that part of the charge carriers will be transported from the perovskite to the germanium layer due to the existence of a large band offset. Benefitting from electron transportation from perovskite to germanium, an enhanced photoconductive gain has also been estimated. The gain is defined as^1,2^ *G* *=* *I*_ph_*/I*_PI_ = *τ/t*_*p*_ *=* *τξ(μ*_*n*_ *+* *μ*_*p*_*)/L*, where *I*_*PI*_ is the primary photocurrent, *τ* is the carrier lifetime, *t*_*p*_ is the carrier transit time across the electrodes, *ξ* is the applied electric field, *L* is the channel width, and *μ*_*n*_ and *μ*_*p*_ are the electron and hole mobility, respectively. Under Vis light excitation, some of the photogenerated electrons are transferred from the perovskite layer to the germanium layer. Compared with perovskite, the ultra-high electron mobility (≈3800 cm^2^ V^−1^ s^−1^) and long carrier lifetime (≈ 200 μs) in the germanium layer result in an enhanced gain of ~10^4^ in our heterojunction photodetector. This is approximately two orders higher than that in the pristine perovskite photodetector. This result indicates a photoconductive gain boost obtained in the heterojunction device. It proves the superior photocurrent (Fig. 5c) and device performance that have been achieved in the Vis light region even with a thinner perovskite layer.

## Discussion

To compare the performance of our work and those reported, the main parameters of the heterostructure broadband photodetectors are summarized in Table 1. Notably, the achieved device in this work shows significant performance promotion compared with previously reported works on the Vis and IR spectra. As discussed above, the reasons and mechanisms behind device performance enhancement are attributed to the perovskite/germanium heterostructure.

**Table 1:** Performance comparison of heterostructure broadband photodetectors.

Device	Wavelength (nm)	*R* (A/W)	D* (Jones)	On/off time (ms)	Refs.
Graphene/Bi_2_Te_3_	532	35	–	8.7/14.8	13
	980	8.5			
	1550	0.22			
Perovskite/MoS_2_	514	68.11	–	0.205/0.206	14
ZnO/PbS	350	51 × 10^−6^	3.4 × 10^8^	<500	15
	500	7.2 × 10^−6^	4.9 × 10^7^		
	900	11 × 10^−6^	7.2 × 10^7^		
Perovskite/ZnO	White light	28	1.1 × 10^12^	0.4/0.5 × 10^3^	16
	1357	0.22	9.3 × 10^9^	0.3/0.8 × 10^3^	
Perovskite/PDPP3T	365	10.7 × 10^−3^	6.1 × 10^9^	–	17
	650	25.5 × 10^−3^	3.2 × 10^9^		
	935	5.5	1.5 × 10^10^		
Perovskite/TiO_2_/Si	350	0.07	5.5 × 10^12^	–	18
	800	0.87	6 × 10^12^		
	1100	0.2	1.2 × 10^12^		
Perovskite	680	230	1.7 × 10^10^	4.2/9.4	This work
Germanium	1550	0.8	9.1 × 10^7^	2.2/5.6	
Perovskite/Ge	405	395	2.8 × 10^10^	4.1/9.2	
	680	228	1.6 × 10^10^	1.8/5.1	
	980	32	2.2 × 10^9^	2.1/5.7	

In this study, we design and construct a heterostructure photodetector successfully by combining inorganic semiconductor germanium with hybrid inorganic-organic perovskite CH_3_NH_3_PbI_3_. A vapor-solution process provides a uniform and pinhole-free perovskite film on a germanium layer. The constructed heterojunction photodetector shows broader bandwidth and enhanced performance compared with those of the single-material-based device. The detection properties of the heterojunction photodetector are characterized at a Vis light wavelength of 680 nm and an optical communication band of 1550 nm. Under Vis light illumination, the free electrons photogenerated in the perovskite are partly transferred to the germanium, resulting in a photoconductive gain boost. The device shows outstanding responsivity and detectivity of 228 A/W and 1.6 × 10^10^ Jones at a wavelength of 680 nm, respectively. When the perovskite AR coating thickness is optimized, the heterojunction device possesses the highest responsivity of 1.4 A/W at an optical fiber communication band of 1550 nm. The germanium/perovskite heterostructure has a broadband detection range from the ultraviolet to the Vis and then to the IR spectrum. Its high performance shows great potential application in wide-spectrum photodetection, ultraviolet-Vis or optical communication, tandem solar cells, and next-generation optoelectronic devices.

## Materials and methods

### Fabrication procedure

The Si coated with SiO_2_ substrates is cleaned by the traditional method. First, a 300 nm Ge is deposited by means of epitaxy onto the cleaned Si substrate using a solid-source MBE system, which is covered by a 350 nm-thick SiO_2_ film deposited by using a plasma enhanced chemical vapor deposition (PECVD) system. This wafer is directly bonded to a handle Si substrate using the benzocyclobutene (BCB) wafer bonding technique, followed by thermal treatment at 260 °C under vacuum for 6~8 h. Then, the initial Si substrate on the Ge film is completely removed by the inductively coupled plasma (ICP) dry etching and tetramethylammonium hydroxide (TMAH) wet etching techniques. Then, gold interdigital electrodes with an electrode width (*W*) and inter-electrode space length (*L*) of 2 and 0.05 mm, respectively, are formed by thermal evaporation. The active area of the heterojunction photodetector is 0.6 mm^2^. A highly crystalline and compact perovskite CH_3_NH_3_PbI_3_ thin film is constructed by a two-step method on the germanium layer. A high-purity and homogeneous PbI_2_ film was first prepared by using a thermally physical vapor phase growth at a pressure of 10^−4^ Pa. The deposition rate and film thickness are monitored by a quartz crystal oscillator. The samples are kept at room temperature during the deposition process. An isopropanol solution of CH_3_NH_3_I (10 mg/ml) is then spin-coated onto the high-quality PbI_2_ films at 3000 rpm for 30 s. Immediately, the samples are moved to a hot plate for annealing at 100 °C for 30 min in an ambient environment. The PbI_2_ and CH_3_NH_3_I powders are purchased from Xi'an Polymer Light Technology Corporation and used without further purification.

### Material and device characterizations

The morphology is characterized by a scanning electron microscope (SEM, Hitachi S-4800, Japan). The performance measurements are all performed in a clean room at a constant room temperature of 23 °C. The current-voltage characteristics are determined on a probe station, and the data are recorded by a semiconductor parameter analyser (Keithley 4200) in the atmosphere. A xenon lamp (PL-SPS1000, Perfect Light Co. Ltd., China) with a monochromatic light source is used as the Vis light source. Two semiconductor lasers (980 and 1550 nm) are used at the IR light source. The incident light power is calibrated before the measurements by a standard silicon photodetector (PM100D, Thorlabs, Germany). The PL spectrum is characterized by confocal microscopy (LEICA DM 2700 M) and recorded by a spectrometer equipped with a CCD and a TCSPC detector (ANDORSR-500i-B1-R). The time-resolved PL spectrum measurements are performed by the TCSPC, in which a picosecond diode laser (*λ* = 405 nm, ≈80 ps, 20 MHz) is used as the excitation source and the overall time resolution is ~250 ps. All measurements are performed at room temperature.

## Supplementary information

Supplementary Informantion
