# Supplementary material for: Germanium/perovskite heterostructure for high-performance and broadband photodetector from visible to infrared telecommunication band
Source: Light Sci Appl. 2019 Nov 21;8:106. doi: 10.1038/s41377-019-0218-y (PMC6872564; doi:10.1038/s41377-019-0218-y)
Supplement: Supplementary file 1 — Supplementary Informantion [file 41377_2019_218_MOESM1_ESM.docx]

**Germanium/Perovskite Heterostructure for High-performance and Broadband Photodetector from Visible to Infrared Telecommunication Band**

*Wei Hu^1^*, *Hui Cong^2,3^*, *Wei Huang^1^*, *Yu Huang*^1^, *Lijuan Chen^1^, Anlian Pan^4^*, *Chunlai Xue^2,3*^*

^1^Key Laboratory for Micro-Nano Physics and Technology of Hunan Province, School of Physics and Electronics, Hunan University, Changsha, Hunan 410082, P. R. China

^2^State Key Laboratory on Integrated Optoelectronics, Institute of Semiconductors, Chinese Academy of Sciences, Beijing 100083, China

^3^Center of Materials Science and Optoelectronics Engineering, University of Chinese Academy of Sciences, Beijing 100049, P. R. China

^4^Key Laboratory for Micro-Nano Physics and Technology of Hunan Province, College of Materials Science and Engineering, Hunan University, Changsha, Hunan 410082, P. R. China

**Figure S1**. The simulation results of the optical filed distribution for the perovskite AR coating layer on germanium. The AR layer thickness are 120 and 180 nm.


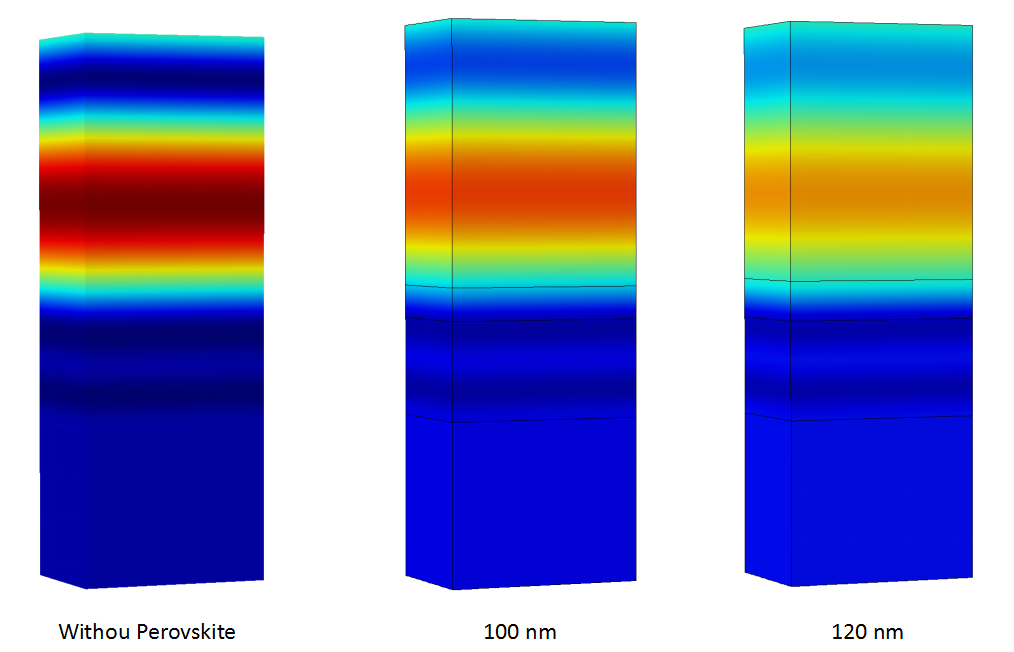

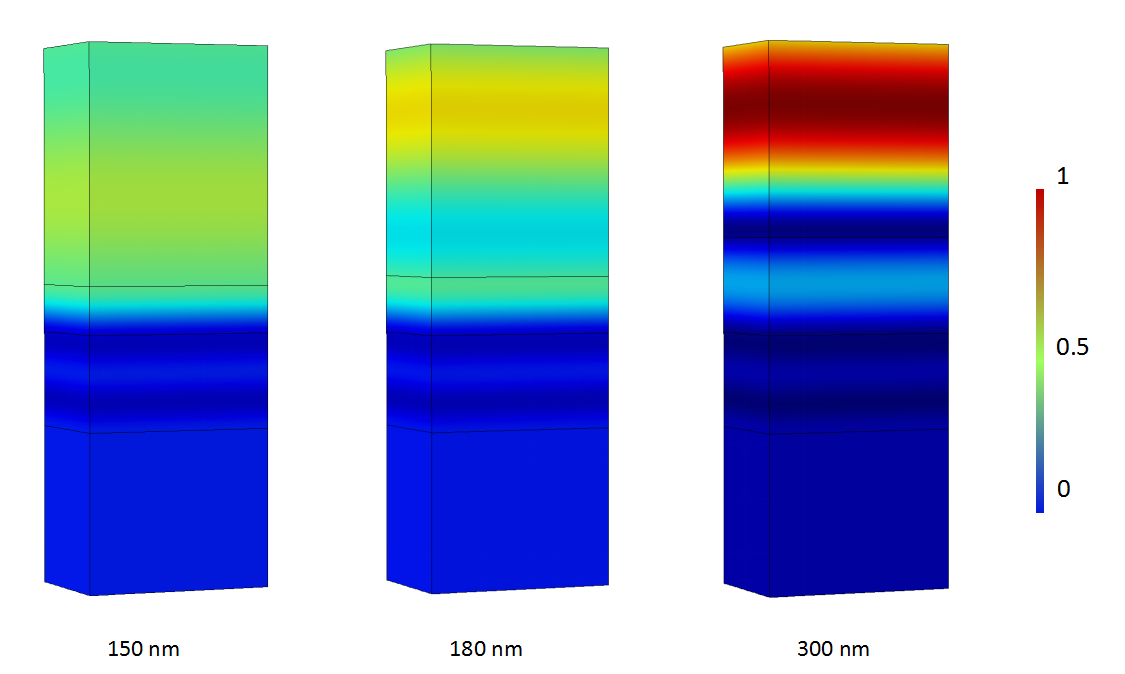

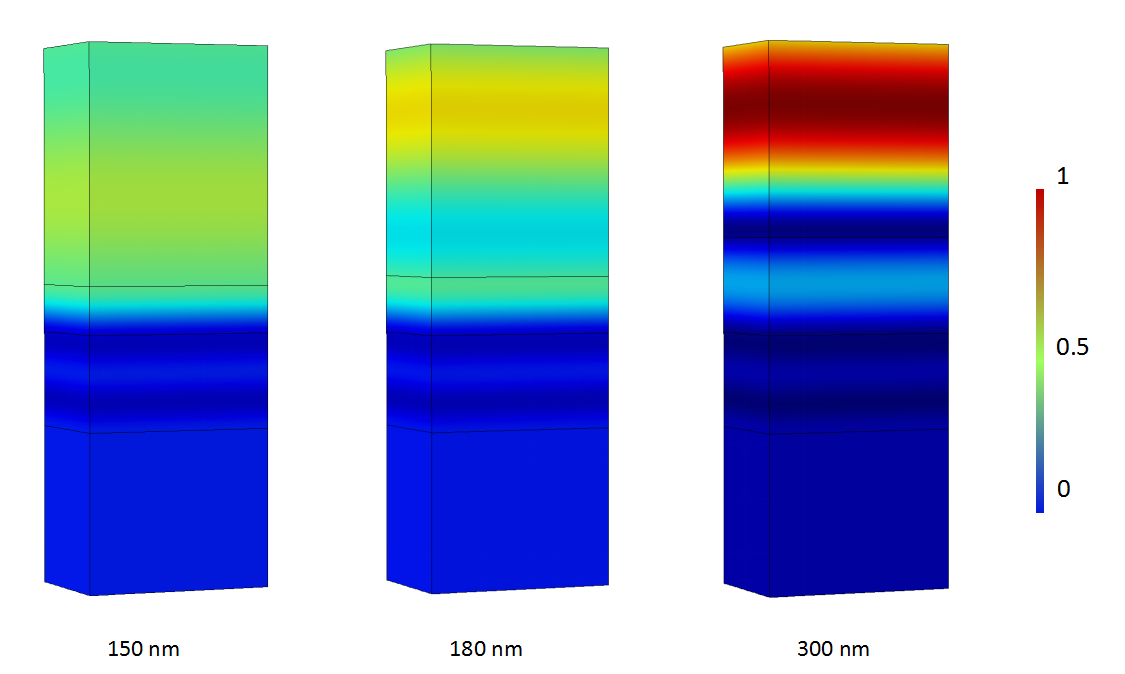


**Air**

**Perovskite**

**Germanium**

**SiO_2_**

**Figure S2**. The reflectance spectrum in the infrared region for the heterojunction device with 150 nm AR coating thickness.


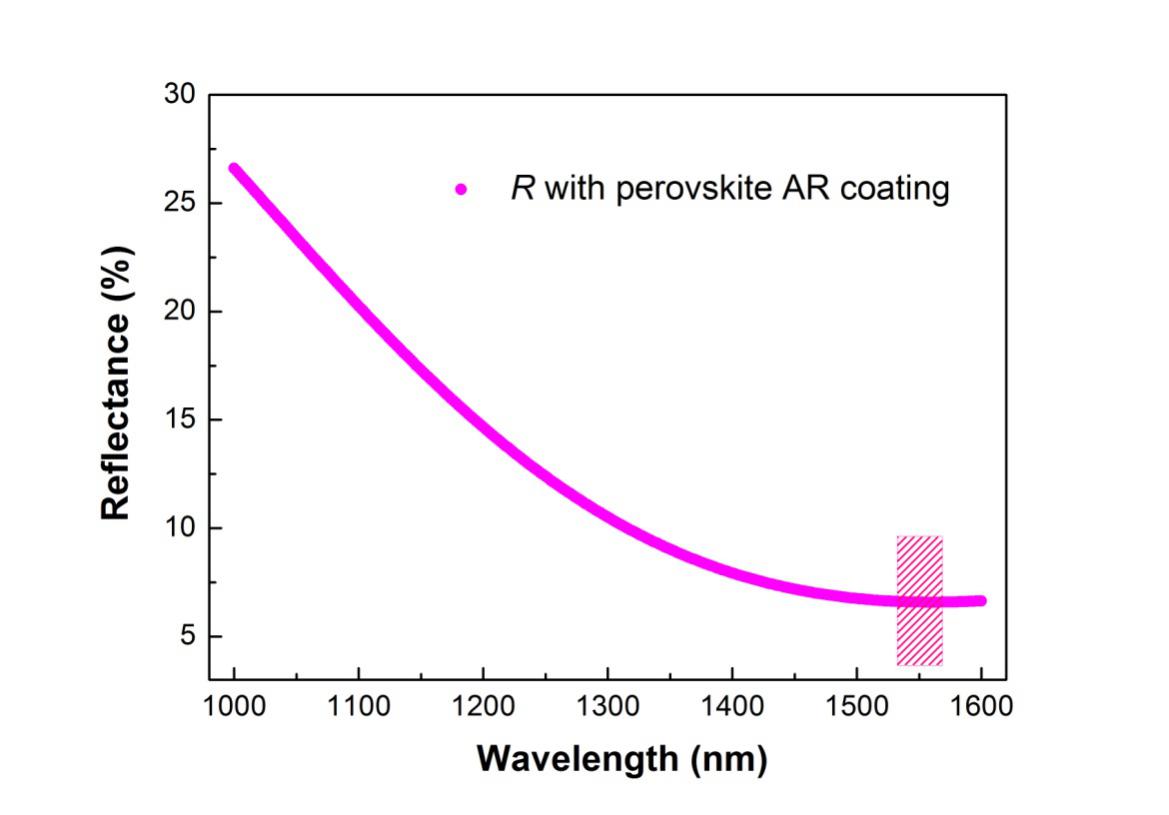


**Figure S3**. The cross-sectional SEM images for the obtained heterojunction devices with different perovskite thickness (scar bar 200 nm).

(a) 100 nm





(b) 120 nm





(c) 180 nm





**Figure S4**. *I-V* curves comparison for the pristine germanium covered with 150 nm AR coating layer at (a) 1550 nm and (b) 980 nm.

(a)


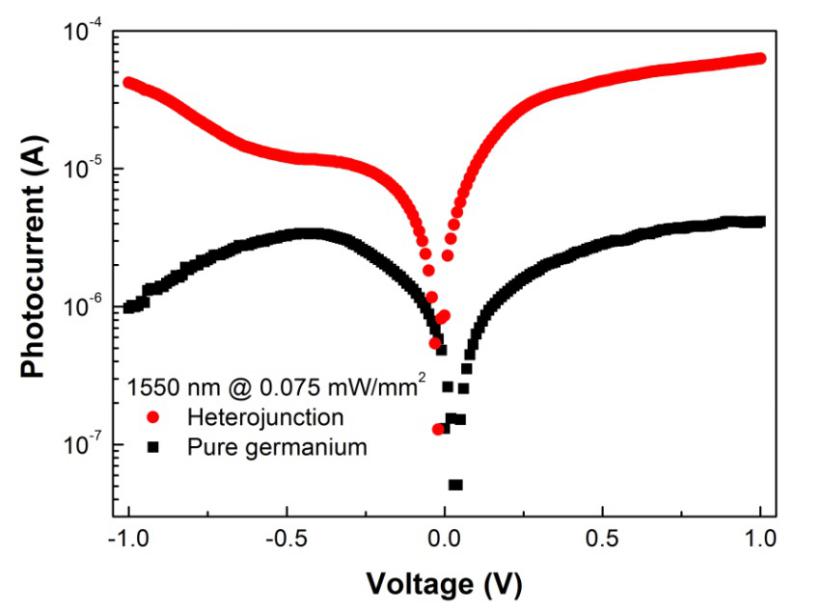


(b)


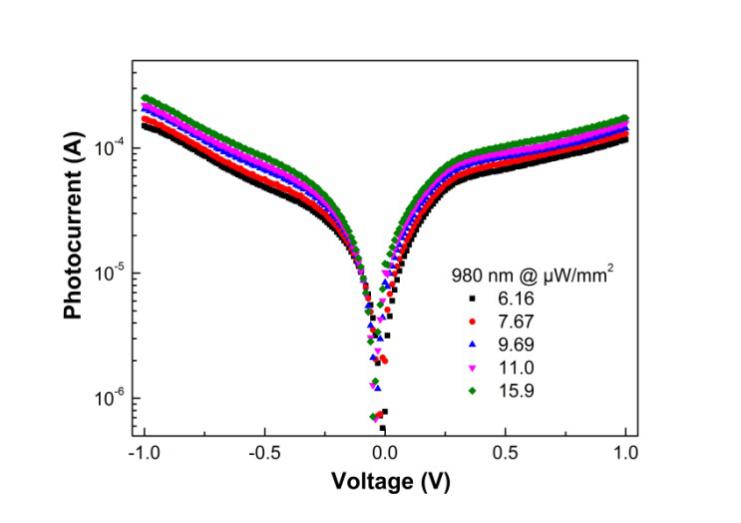


**Figure S5**. The noise currents of the pristine germanium device at different frequencies.


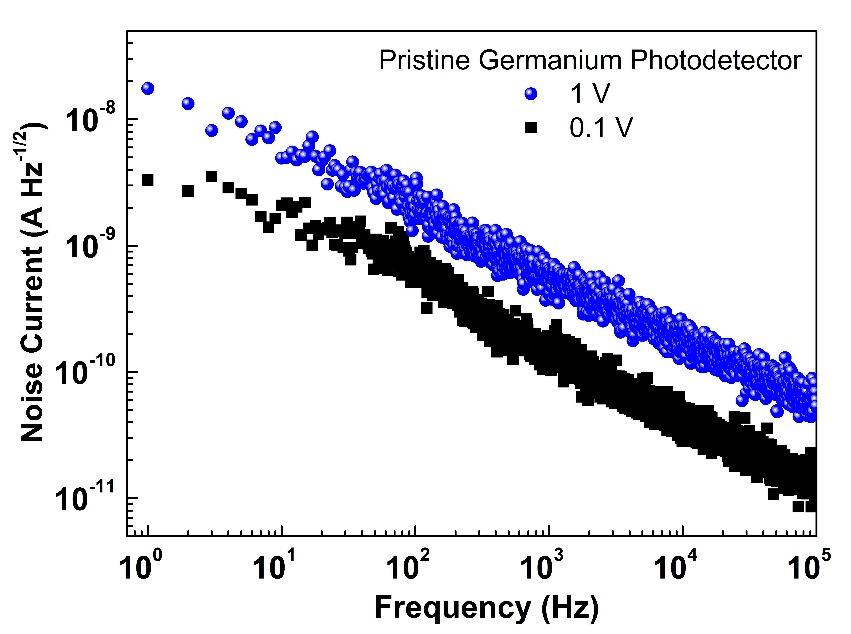


**Figure S6**. *I-V* curves comparison for the pristine germanium and 150 nm AR coating layer heterojunction photodetector at 450 and 680 nm.


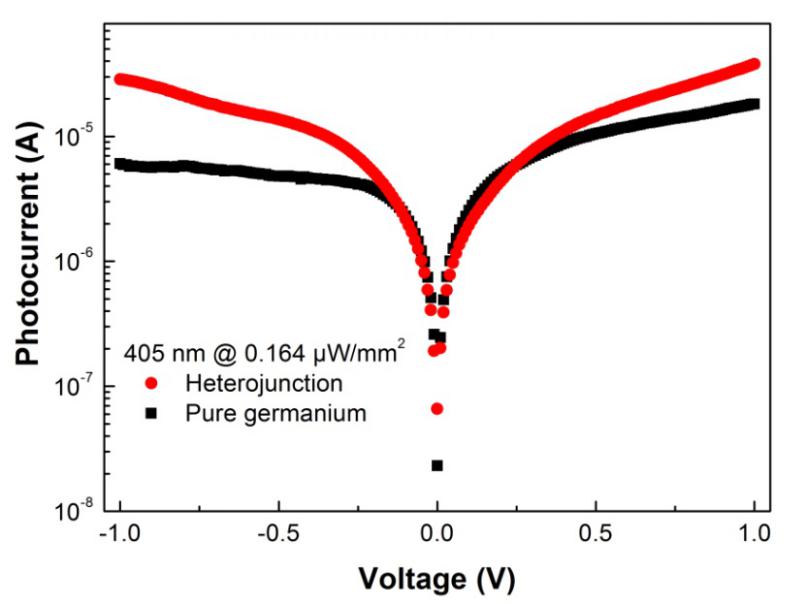


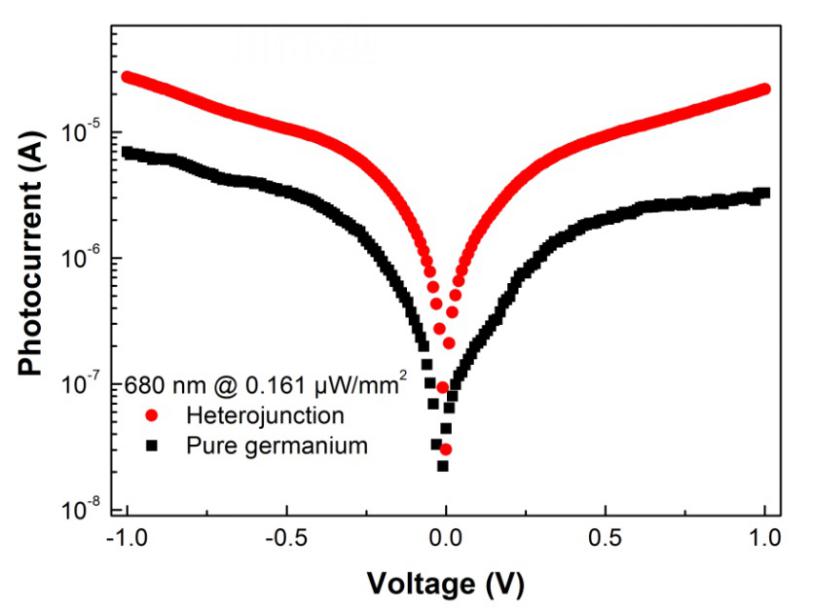


**
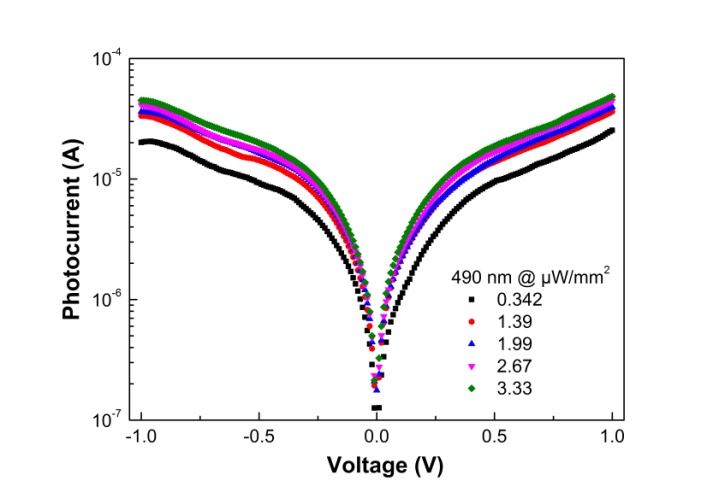

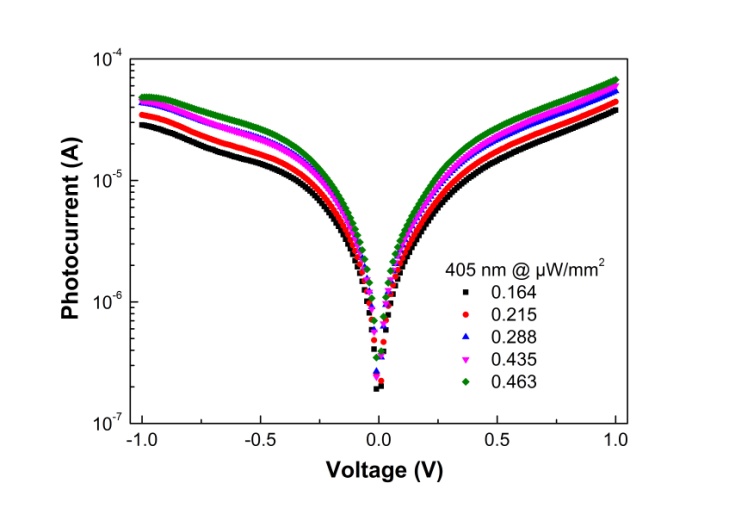
Figure S7**. *I-V* curves of the optimized heterojunction photodetector under different incident light wavelength (405, 490, 590, 680 nm) and power density.


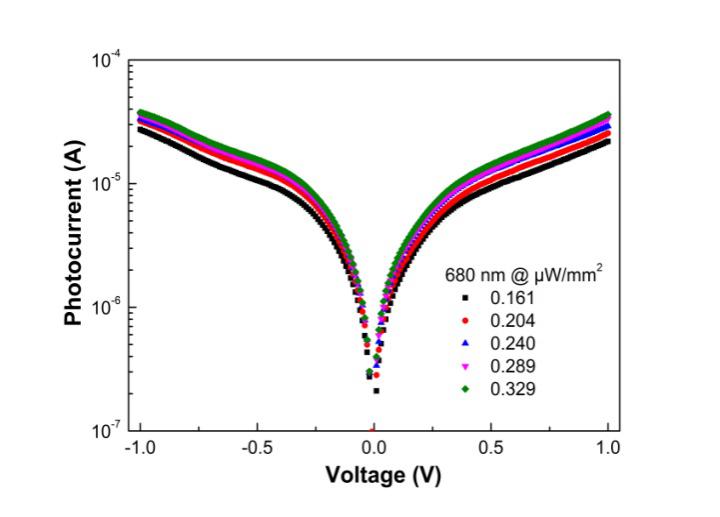

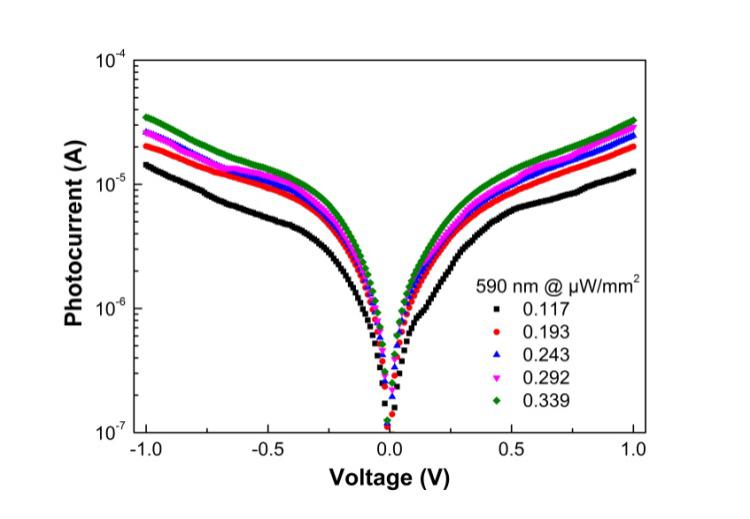


**Figure S8**. The UPS spectra of the perovskite and germanium layer.


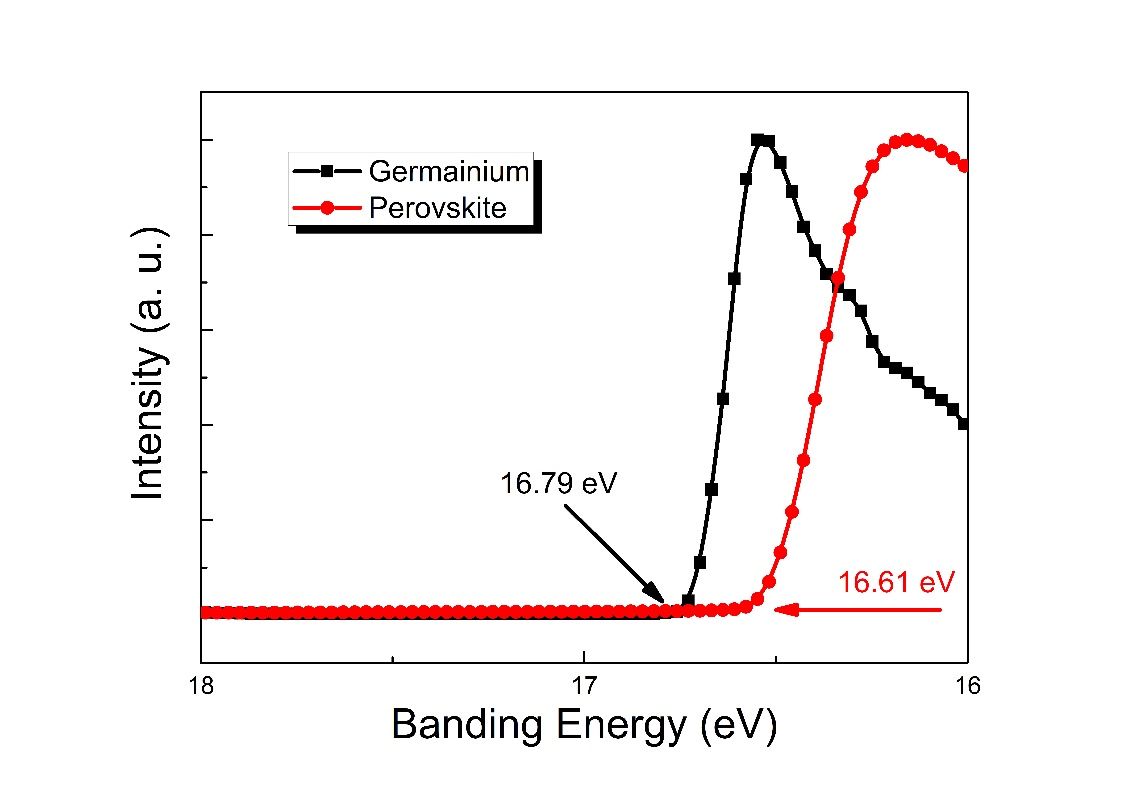


The work function of the two materials are calculated below:

*W*(germanium) = 21.22 – 16.79 = 4.43 eV

*W*(perovskite) = 21.22 – 16.61 = 4.61 eV

The work function difference between the perovskite and germanium in the heterojunction is
*ΔW* = *W*(perovskite) - *W*(germanium) = 0.18 eV

**Table S1.** The carrier lifetime results.

|  | Heterojunction | error | Pristine | error |
| --- | --- | --- | --- | --- |
| τ_1_ | 11.5 | ±0.1 | 19.0 | ±2 |
| *τ_2_* | 2.8 | ±0.1 | 6.1 | ±0.2 |

The charge carrier lifetime fitting process based on the PL decaying experiment. The lifetime was fitted based on the exponential decaying equation SE 1.

$y=y_{0}+A_{1}e^{(-x/t_{1})}+A_{2}e^{(-x/t_{2})}$ (SE 1)
